# Material Flow Analysis of Fossil Fuels in China during 2000–2010

**DOI:** 10.1100/2012/625828

**Published:** 2012-12-30

**Authors:** Sheng Wang, Jing Dai, Meirong Su

**Affiliations:** ^1^Chongqing Academy of Social Sciences, Chongqing 400020, China; ^2^State Key Joint Laboratory of Environmental Simulation and Pollution Control, School of Environment, Beijing Normal University, Beijing 100875, China

## Abstract

Since the relationship between the supply and demand of fossil fuels is on edge in the long run, the contradiction between the economic growth and limited resources will hinder the sustainable development of the Chinese society. This paper aims to analyze the input of fossil fuels in China during 2000–2010 via the material flow analysis (MFA) that takes hidden flows into account. With coal, oil, and natural gas quantified by MFA, three indexes, consumption and supply ratio (C/S ratio), resource consumption intensity (RCI), and fossil fuels productivity (FFP), are proposed to reflect the interactions between population, GDP, and fossil fuels. The results indicated that in the past 11 years, China's requirement for fossil fuels has been increasing continuously because of the growing mine productivity in domestic areas, which also leads to a single energy consumption structure as well as excessive dependence on the domestic exploitation. It is advisable to control the fossil fuels consumption by energy recycling and new energy facilities' popularization in order to lead a sustainable access to nonrenewable resources and decrease the soaring carbon emissions.

## 1. Introduction

Material flow analysis (MFA), quantified by material weight instead of currency, is built on the theories of industrial metabolism and social metabolism to pursue the translation path from nature to human ecosystem as well as the final regressive sinks [1]. The basic standpoint of MFA is that environmental effects which are brought about via social economic behaviors are mainly dependent on the quantity and quality of natural resources and materials devoted in the ecosystem, and the wastes translated from the consumption sectors back to the environment.

MFA is viewed as a significant measure to study the metabolism of material and resources. Ayres and Kneese firstly presented the first material flow accounts on the national level in 1969 [2]. During 1970s–1980s, the perfection of material balance and industrial metabolism theories laid a solid foundation for MFA application in the whole ecosystem. Austria, Japan, and Germany took the lead in calculating substance and natural resources in domestic economic scope in 1990s [3]. Subsequently, developed countries such as The Netherlands, America, and Australia finished their MFA in national boundary [1, 4]. In 2001, EU Statistics Department published a handbook about MFA research technique applied to ecosystem for the first time in the world [5], which enormously accelerated the promotion of MFA in economic field. Muñoz and Hubacek pointed out in 2008 that the economic growth was the major source of material changes [6] Chen proposed in his study in 2007 that the driving force of the social-economic-ecological complex system was the resource, which posed unparalleled challenges on each level of the society. The quantity and quality scarcities of the diverse resources require an efficient, effective, and interdependent utilization based on overall and unified accounting [7]. In recent years, China has also done necessary studies in MFA and gained achievements in scientific research of the relationship between total material input and consumption in the national base [8–10].

In the early 1990s, The concept of “ecological rucksacks” was firstly proposed which was commonly accepted as “hidden flows” afterward [11]. This concept refers to the wastes inevitably produced in the process of resource exploitation, though it is not devoted to the social production. Without creating commercial value, it will exert a huge influence on natural and social environment. In view of this, MFA is further modified and becoming an effective tool in measuring the balance between the resource depletion and the social development [12].

In this study, MFA's method is applied to the Chinese fossil fuels as a research case with certain modifications of hidden flows, which is considered as the more exact mode in accounting resource consumption. The results can make acceptable recommendations not only in transferring materialized into dematerialized consumption pattern, but also in building a low-carbon economy and coping with global warming.

## 2. Methodology and Data

The study period, lasting from 2000 to 2010, was an important stage for China's rapid economic progress in history. The rapid growth in the fields of both economy and resource exploitation is producing far-reaching impacts in social and environmental areas for today's decision making and stratagem implement. The basic information of China during the study period is listed as background information in Table 1.

Currently, a systemic MFA framework based on the national or regional ecosystem has been initially established all over the world, which has been comprehensively applied in occidental countries. The input stream of fossil fuels was divided into two parts, direct input flows, and hidden flows. The study boundary was confined into the domestic ecosystem. The input fossil fuels which come from the domestic production and abroad import contain raw coal, oil, and natural gas, excluding secondary energy input. Moreover, Wuppertal evaluated the average ratio of global hidden flow (GHF), and the results showed that crude oil, natural gas, and raw coal were 1 : 1.22, 1 : 1.66 [13], and 1 : 2.36 [14], respectively in view of the fact that most coal in China belongs to bastard coal. Usually the output stream of fossil fuels refers to direct output and contaminated discharge. Due to different burning efficiencies and regional variation in technologies, it is inaccurate to calculate direct output at the end of MFA. To express the end-result of the fossil fuels in the whole process of material flow, this paper chose three indexes, consumption and supply ratio (C/S Ratio), resource consumption intensity (RCI), and fossil fuels productivity (FFP) by considering population, GDP, and total energy input, to evaluate the influences of resource consumption in the fields of society and economy as well as environment. The three indexes are defined as follows:

(1) $$\begin{array}{c} \frac{\text{S}}{\text{C}}=\frac{\text{Consumption}}{\text{Production}-\text{Export}+\text{Import}}\times 100\%,\\ \text{RCI}=\frac{\sum \text{Consumption}\times \text{GHF}}{\text{Population}},\\ \text{FFP}=\frac{\text{GDP}}{\sum \text{Consumption}\times \text{GHF}}, \end{array}$$

in which, the units are tsc/person (“tsc” refers to ton of standard coal (we use tsc for short in the rest part of this paper); standard coal, also known as coal equivalent, unified calorific value standard, different varieties, different energy contents of different calorific values converted to the calorific value of 7,000 kcal per kg of standard coal) for RCI, $/tsc for FFP (it is better for further comparison with other countries when we replace the currency unit from “¥” to “$”).

All the data sources are acquired from publications, such as Energy Statistical Yearbooks of different years [15] and China Statistical Yearbooks [16] and websites [17]. In order to seek unity of economic value and avoid inflation or deflation in different times, we defined the year of 2000 as a base year, in which year the price was chosen as constant price; therefore, pure monetary value in all the other years should be converted into a standard value of 2000. The original yield and import/outport flows of three primary fossil fuels are listed in Table 2.

According to IPCC 2006 [18], different types of fuels have their own carbon emission coefficients (we use CEC in the following part of this paper), for coal, oil, and natural gas, the transition factors are listed in Table 4. Therefore, the CO_2_ emission distributions will be obtained on the basis of fossil energy structure.

## 3. Results and Discussion

C/S ratio is of a paramount importance for the national or regional sustainability. A high value (>1) of C/S ratio means more resources will be depleted, and then more wastes or disturbuances will occur along with environmental deteriorations and nonrenewable energy shortage. If the C/S ratio is low, on the one part, it is good news that the present fossil energy supply is sufficient for demand. On the other part, it provides an important implication to readjust the energy consumption and supply relationship in the national level. The index called RCI can reflect the personal access to fossil resource in China. FFP denotes the transfer ability from raw fossil material occupation into economic value, which usually represents the efficiency of the resource consumption. The accounting of MFA and different indexes from 2000 to 2010 is shown in the following table (RMB exchange rate against the US dollar to compare this quota after conversion with different countries in a unified standard), and all the material flows are measured by the unit of  “tsc,” and the fossil fuels' consumption for three types of energy are collected in Tables 3(a) and 3(b).

### 3.1. Diversification of C/S Ratio See Figure 1

During the years from 2000 to 2007, it was nearly stable between 1 and 1.05 implying that a certain amount of previous fossil stock should be used to solve the supply and demand difference. During this period, there was a minor peak in 2003, reflecting that the obvious expansions of real estate construction, iron, and cement based heavy industry soaring development, which lead to the remarkable tension for energy supply and consumption relation. And after 2008, this ratio is fluctuate by most time lower than 1, which mainly attribute to the implementation of “energy saving and emission reduction” strategies in industries and domestic using. Generally speaking, from 2000 to 2010, C/S ratio of fossil fuels was decreasing roughly and the energy balance was trending into a capable supply state. Meanwhile, to better control the C/S ratio is an effective device to control the energy balance in the original production and final consumption.

Considering three kinds of fuels, coal yield was the main growth factor, which indicated that the energy structure in China was mainly based on raw coal as the dominant sector. Moreover, coal domestic production had occupied a share ranged 73–77% compared, and for oil was around 20% with total fossil energy during the past 11 years. The coal dominated energy structure has not dramatically reversed in that period. On average, the requirement of oil in occident countries account for 35% [11] of the total energy, that means we have a long way to amend the energy consumption structure, especially adding new energy into consideration, such as wind power, bioenergy, and tidal energy. Oil supply in China will not be sufficient forever, an urgent support policy is needed to encourage powerful companies and organizations to blend in the international arena and exploit a high quality resource. Furthermore, the domestic natural gas yield as well as the import proportion was enlarging, which indicated a good transition in enriching energy diversity and equitability, but with a too slight step. Therefore, we still have an adequate space to optimize the energy allocation so as to change the irrational existing state of fossil fuels consumption.

### 3.2. Diversification of RCI

RCI is an indicator that can express the level of per capita resource possess.  Table 5 indicates that RCI was 2.1 times in 2010 than that in 2000, which implied that as individuals grow more affluent, their demands move beyond basic needs. The enormous requirements for private car, energy-consumption electronic products made life quality improve deeply on the one hand, and, on the other hand, extremely aggravated fossil resources depletion. The only solution to avoid energy crisis, especially for nonrenewable energy, is to elevate utilization efficiency and attempt new renewable energy. In addition, China is the most populous developing country, whose contradiction between large energy-needed population and low per capita energy possession is always restricting the whole national development in social and economic fields. In addition, CEC from different types of the fossil fuel consumption from 2000 to 2010 in China is shown in Table 6 as a supplementary database.

### 3.3. Diversification of FFP

FFP, an economic value produced by the unit natural nonrenewable resource.  Table 7 clearly illuminates that an obvious disparity is existing in fossil fuel productivity compared with the major energy-saving country. The level of fossil fuel productivity in China in 2010 was merely equivalent to that of The Netherlands in 1996, and Austria, Germany, and Great Britain had already owned this index nearly 1.5 times higher than that of China in 2010. The bog standard of FFP in China means not only a colossal waste of fossil fuels in production and consumption process, but also severe environmental pollution resulting in a great number of pollutants and wastes in all processes by using natural resources. The evident gap between China and other developed countries in creating economic value per unit fossil resource reflected low efficiency in energy input-output course. It is well accepted that economic development based on the extensive resource depletion is not the intrinsic pursuit of sustainable developing pattern, and it is needed to consummate a resource recovery system as soon as possible, promote the use of energy saving products in public, and participate in international cooperation that can develop bilateral and multilateral as well as regional collaboration in the field of new energy using and low-carbon technology [19]. Another step to solve the inefficiency in fossil fuel productivity is to change the approach of using fossil fuels into a closed material cycle mode [20] and enhance the GDP value created by per unit energy expending.

### 3.4. Comprehensive Analysis about Different Indexes

**Figure 2:**
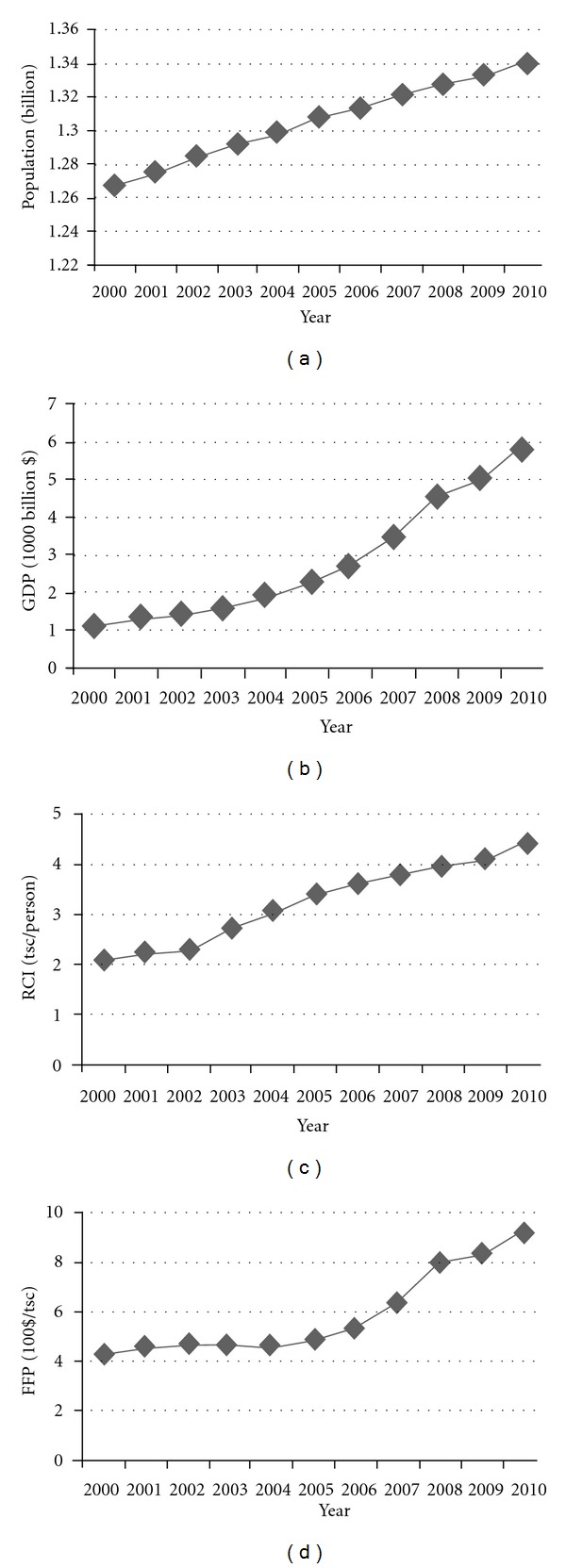
A comprehensive index comparison of fossil fuels in China during 2000–2010.

The variation trends of population, GDP, and RCI as well as FFP are illustrated in Figure 2. First, the population increased steadily which was fitting in with the national conditions during that time; second, GDP increased with an accelerating trend, which was not only due to the economic dynamic times in this period, but also affected by the appreciation of RMB since 2005; third, RCI was increased especially after the year 2002, meaning a society with the rapid consumption for fossil fuels; and finally, the variation of FFP was divided into two subperiods: the first half was nearly fair, however, the other half was soaring for a rapid fossil fuel economic productivity. Generally speaking, economy in China is operating well, but the extensive mode of growth could not change at once, with fossil fuel productivity impossibly boosted in a short time. However, infrastructures on fossil energy being or having been built will certainly carry weight in both economic development and the resource consumption.

### 3.5. CO_2_ Emission Analysis

**Figure 3:**
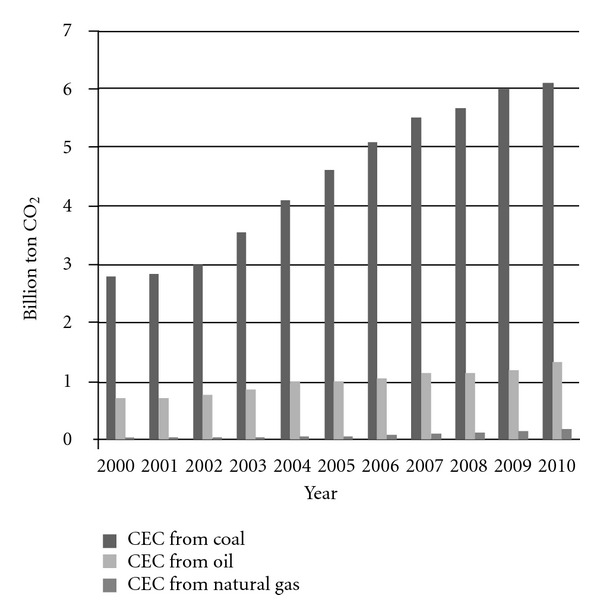
CEC amount from three types of fossil fuels from 2000 to 2010 in China.

From Figure 3, it was clearly demonstrated the CO_2_ emission distribution, and coal is the most carbon source. In China, the hidden flow ration for coal was the highest, and the energy structure could not be changed on the basis of coal consumption. That is why CEC from coal was nearly 2 times than that from oil and natural gas. From this result, using natural gas or the other low carbon emission energy is the most effective and urgent strategy for the government decision and public choice.

## 4. Conclusion

This paper analyzes the input of fossil fuels in China during 2000–2010 and proposed several indicators of MFA. In comparison with other countries, main conclusions are summed up as follows.

Generally speaking, the demand structure of coal, oil, and natural gas during the past 11 years has not changed significantly. The turning point of fossil fuel productivity appeared in 2002, and increased dramatically after 2005. Both phenomena are decided by international economic situations and domestic production levels. The former is due to good circulations of the world economy and China's entry into WTO, which lead to substantial materials input but a lack of efficiency advancements. The latter is owing to policy steering of the eleventh five-year plan unveiled in 2005, which drives the movement of technical improvement, renewable energy development, wastes recycling, and multilevel using. But we have to admit that China still has to go a long way to pursue fossil fuel productivity compared with countries like Japan and Germany.

MFA accounting system calculates environmental pressure produced by the resource depletion. Its standpoint is payments for import only cover the economic costs without environmental costs as well as hidden flows value in the process of energy production. By this token, one country can pass over the environmental cost by importing so as to reduce the domestic natural risk. From this point of view, it is necessary to shift energy strategy from export-oriented to import-oriented style and elevate the export price by considering environmental expense and enlarging import proportion, from which home resource and environment can be protected in a qualified sense. Nevertheless, it is myopia for a particular developing period in certain regions, but not beneficial for universal sustainable utility of resource. Natural wealth belongs to human beings, without boundary of different nations and territories.

## 5. Discussions and Prospects

For the existing energy supply and demand status quo, more efforts are needed to intensify energy structure reforms, though the expansion of gas domestic output and import quotation provided a new perspective. Furthermore, the unchanged conditions may be related to the existing exploitation patterns, the history of coal mining and technical problems in the development of pelagic natural gas, which are primarily reasons for the univocal energy current situation. Whereas, the future structure of energy utility will move forward to a rational and sustainable direction as long as technical progress, sufficient facilities, financing of safeguards, and national supporting policies are provided. Meanwhile, the “closed material cycle mode” that with the process of “energy production → energy consumption → recycled energy → recycled energy reuse” should be extensively implemented in extensive energy based industries in order to decrease the C/S ratio value and create more economic value by consuming the previous amount of energy consumption. And the bundled infrastructures or strategies should be followed up, for example, the generalization of garbage classification with recycled waste energy appliance, popularization of new energy vehicle service facilities.

The transformation of GDP has necessary connections with the resource consumption intensity and the fossil fuel productivity. To accomplish the target of energy saving and emission reducing, and also to keep to the path of sustainable development, it is necessary to rely on domestic research and developments as well as global introductions of advanced technology in enhancing efficiency and reduced consumption for internal upswing.

Apparently, material and energy streams are dependent with each other in a different manner, so the simplex material flow analysis is not comprehensive in identifying the energy consumption in an environmental economic system. Next, we should combine the material and energy analysis to understand the social metabolism of fossil fuels from all angles. At the same time, the indexes and their variation trends provide reasonable explanations for future policy drafts in energy saving and emission reducing by evaluating the recent 11 years' resource consumption and production efficiency.

## Figures and Tables

**Figure 1:**
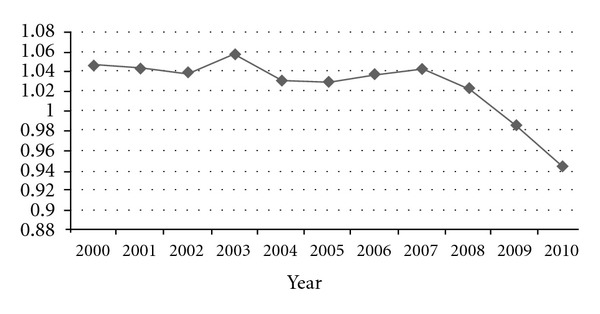
C/S ratio of fossil fuels in China during 2000–2010.

**Table 1:** Population and GDP information of China during 2000–2010.

Year	Total population (million)	Gross domestic product (¥ billion)
2000	1267.43	9921.5
2001	1276.27	10965.5
2002	1284.53	12033.3
2003	1292.27	13582.3
2004	1299.88	15987.8
2005	1307.56	18308.5
2006	1314.48	21087.1
2007	1321.29	24953.0
2008	1328.02	31404.5
2009	1334.50	34090.2
2010	1340.91	40120.2

**Table 2:** The original yield and import/outport flows of three primary fossil fuels during 2000–2010.

Year	Coal			Oil			Natural gas		
	Yield	Export	Import	Yield	Export	Import	Yield	Export	Import
	(million tsc)	(million ton)	(million ton)	(million tsc)	(million ton)	(million ton)	(million tsc)	(billion cu.m)	(billion cu.m)
2000	988.55	55.06	2.17	232.28	10.30	70.26	36.46	3.1	0
2001	1050.28	90.12	2.66	234.51	7.55	60.26	40.28	3.0	0
2002	1107.32	83.89	11.25	238.03	7.66	69.40	43.69	3.2	0
2003	1309.92	94.02	11.09	242.38	8.13	69.40	46.41	1.8	0
2004	1516.15	86.66	18.61	2517.01	11.46	122.72	55.06	2.4	0
2005	1677.85	71.68	26.17	259.46	8.07	126.82	64.86	2.9	0
2006	1806.25	63.23	38.25	262.34	6.34	145.18	78.93	2.9	1.0
2007	1921.35	53.17	51.01	267.06	3.98	163.17	91.49	2.6	4.0
2008	2001.03	45.43	40.40	273.57	4.16	178.88	106.56	3.2	4.6
2009	2122.80	22.40	125.83	271.87	5.07	203.79	112.59	3.2	7.6
2010	2271.40	19.03	164.78	290.97	3.03	239.31	127.67	4.0	16.5

**Table:** (a)

Year	Coal	Oil	Natural gas	Total consumption
2000	1007.07	323.08	32.02	1362.17
2001	1027.27	3278.89	36.10	1391.26
2002	1084.13	355.53	38.26	1477.93
2003	1282.87	389.64	45.95	1718.46
2004	1483.52	454.66	53.36	1991.54
2005	167.08	467.27	61.36	2199.49
2006	1839.19	499.24	75.02	2413.45
2007	1994.41	527.36	92.57	2614.33
2008	2048.88	533.35	107.84	2690.07
2009	2158.79	548.90	119.59	2827.29
2010	2209.59	617.38	142.97	2969.94

**Table:** (b)

Year	Coal	Oil	Natural gas	Total
2000	950.77	317.94	32.65	1301.37
2001	987.81	309.82	36.60	1334.22
2002	1055.44	326.24	39.81	1421.48
2003	1250.69	329.92	44.14	1624.75
2004	1467.55	410.66	52.10	1930.30
2005	1645.35	429.11	61.26	2135.72
2006	1788.42	460.70	76.57	2325.68
2007	1919.81	494.48	93.22	2835.45
2008	1997.45	523.18	108.20	2628.83
2009	2196.68	555.76	117.96	2870.41
2010	2375.52	628.53	142.77	3146.82

^2^Supply = production − outport + inport.

**Table 4:** The carbon emission coefficients (CEC) for three types of fossil fuels.

Items	CEC
Coal	1.98 ton CO_2_/ton
Oil	3.07 ton CO_2_/ton
Natural gas	2.19 ton CO_2_/ton

**Table 5:** C/S ratio, RCI, and FFP of China during 2000–2010.

Year	C/S ratio	Population	RCI	GDP	Exchange rate	GDP	FFP
		million	(tsc/person)	(¥ billion)	(¥/100 $)	($ billion)	($/tsc)
2000	1.0467	1267.43	2.11	9921.4	827.84	1198.46	424.39
2001	1.0427	1276.27	2.22	10965.5	827.70	1324.82	459.32
2002	1.0397	1284.53	2.32	12033.2	827.70	1453.82	475.76
2003	1.0577	1292.27	2.68	13582.2	827.70	1640.97	458.47
2004	1.0317	1299.88	3.059	15987.8	827.68	1931.64	466.09
2005	1.0299	1307.56	3.35	18493.7	819.17	2257.62	489.17
2006	1.0377	1314.48	3.59	21631.4	797.18	2713.50	534.76
2007	1.0426	1321.29	3.79	26581.0	760.40	3495.67	635.13
2008	1.0233	1328.02	3.94	31404.5	694.51	4521.83	798.20
2009	0.9850	1334.50	4.14	34090.2	683.10	4990.53	836.92
2010	0.9438	1340.91	4.42	40120.2	696.95	5756.54	927.70

**Table 6:** CEC from different types of the fossil fuel consumption from 2000 to 2010 in China.

Year	Coal consumption	Oil consumption	Natural gas consumption	CEC from coal	CEC from Oil	CEC from natural gas
	(million ton)	(million ton)	(million ton)	(million ton CO_2_)	(million ton CO_2_)	(million ton CO_2_)
2000	1409.87	226.15	18.92	2791.55	694.28	41.45
2001	1438.15	229.51	21.34	2847.54	704.61	46.73
2002	1517.75	248.87	22.62	3005.15	764.02	49.54
2003	1795.97	272.74	27.16	3556.03	837.31	59.49
2004	2076.88	318.26	31.55	4112.23	977.04	69.09
2005	2339.15	327.09	36.27	4631.52	1004.15	79.44
2006	2574.80	349.46	44.35	5098.12	1072.85	97.13
2007	2792.12	369.14	54.72	5528.39	1133.26	119.85
2008	2868.37	373.33	63.75	5679.38	1146.14	139.62
2009	3022.25	384.22	70.70	5984.05	1179.55	154.84
2010	3093.35	432.16	84.53	6124.84	1326.73	185.12

**Table 7:** Statistics of FFP in other countries during recent years (Unit: $/tsc).

Country	1975	1980	1985	1990	1995	1996
Austria	649.9	887.8	620.3	1197.9	1415.7	1362.2
Germany	649.8	984.6	751.6	1451.6	1296.6	1313.9
Japan	472.1	700.6	852.8	1410.2	2543.3	2255.7
Netherlands	460.5	670.9	437.1	884.2	1018	949.1
Great Britain	1487.01	1714.01	1012	1517.1	1482.2	1513.2
